# Conflict between conservation and development: cash forest encroachment in Asian elephant distributions

**DOI:** 10.1038/s41598-017-06751-6

**Published:** 2017-08-03

**Authors:** Peng Liu, Hui Wen, Franziska K. Harich, Changhuan He, Lanxin Wang, Xianming Guo, Jianwei Zhao, Aidong Luo, Hongpei Yang, Xiao Sun, Yang Yu, Shaobo Zheng, Jing Guo, Li Li, Li Zhang

**Affiliations:** 1 Key Laboratory for Biodiversity Science and Ecological Engineering, Ministry of Education, College of Life Sciences, Beijing Normal University, Beijing, 100875 China; 2 College of Urban and Environmental Sciences, Peking University, Beijing, 100871 China; 3 Institute of Plant Production and Agroecology in the Tropics and Subtropics, University of Hohenheim, Stuttgart, 70599 Germany; 4 Research Institute of Xishuangbanna National Nature Reserve, Jinghong, Yunnan 666100 China; 5 Wildlife Management and Ecosystem Health Center, Yunnan University of Finance and Economics, Kunming, 650221 China

## Abstract

Over the last 4 decades, China has undergone major economic development, resulting in considerable impacts on its wildlife populations and habitats. It is essential to quantify the conflict between development and conservation to assist with policy-making because forestry policies and market trends affected indirectly the distribution of Asian elephants. Here, we mapped the historical distribution of elephants versus human land use. Elephant distributions appear to occur in unbroken natural forests only. However, over the 40-year period, the distribution ranges have become smaller and fragmented, with natural forest area also declining by 16%. The monoculture of cash trees is encroaching on natural forests. Over the past 10 years, rubber plantations have become concentrated in the south, with extensive natural forests and scattered rubber farms being converted to tea plantations, due to changes in governmental policies and product prices. Through mapping the spatial changes in the distribution of rubber and tea plantations, our study is expected to help local managers to incorporate the needs of endangered elephants through creating space when planning plantations, especially in Xishuangbanna and the south part of Pu’er. In conclusion, restoring elephant habitat and establishing ecological corridors are critical for the survival of elephants in this region.

## Introduction

Approximately 6,000 years ago, Asian elephants *Elephas maximus* were found throughout western Mesopotamia, the Indian subcontinent, Southeast Asia, and the eastern Yangtze River and Yellow River basins of China^1^. With the development of human civilization, the distribution of Asian elephants has dramatically decreased, and they have disappeared from West Asia, Persia, Java, and most parts of China^1^. Surveys in the 1950s confirmed that elephants were still present in the southern Yunnan Province of China^2^. Subsequent studies in the 1960s indicated that Asian elephants were distributed in the Jinghong and Mengla counties of Xishuangbanna, Ximeng County of Pu’er, Cangyuan County of Lincang, and Yingjiang County of Dehong in China^3^. However, these populations gradually disappeared in the 1980s and 1990s^3^ because of poaching and economic development.

Habitat loss and illegal poaching threaten the survival of Asian elephants^4^, with elephants currently occupying only 5% of their original habitat^5^. Indeed, recent studies have indicated that only 200–250 Asian elephants inhabit Pu’er City, Xishuangbanna National Nature Reserve and Nangunhe Nature Reserve^6–8^. Agriculture is a major cause of habitat loss^9, 10^, negatively affecting the distribution of wildlife^11–13^, including Asian elephants^14^. Protected areas (PAs) are often too small and insular to support Asian elephants that cannot migrate to other areas, due to the expansion of plantations^15^.

The relative value of natural forests versus farmland is always considered a thorny issue. Natural forests are the preferred habitat of Asian elephants^8, 16, 17^. Previous studies showed that the observed frequency of elephants in natural forests, especially tropic rainforests, is higher than in farmland^18, 19^. While some elephants have been recorded using farmland, it had always been regarded as a secondary component of the foraging habitat and migration pathway of elephants, rather than true suitable habitat^20, 21^. In the last four decades, rubber and tea plantations have been used as an effective approach to reduce poverty in Asia. In fact, the displacement of natural forest due to the expansion of plantations led to increased crop raiding by elephants, and severe human-elephant conflicts^22, 23^.

To address the concern that plantations have encroached on existing natural forests and protected areas (PAs), the Central Government initiated two major conservation policies in the late 1990s: the Natural Forest Conservation Program (NFCP) and the Sloping Land Conservation Program (SLCP). The Yunnan Provincial Government advocated increased funding to expand conservation areas in 2007^24^. In addition, the Xishuangbanna Prefectural Government and rubber industry established the “Leadership Group for Environmentally Friendly Rubber” (LGEFR) in 2009^25^. Yet, the natural rubber market collapsed a few years ago, whereas the Pu’er tea industry prospered after 2003. We speculate that this shift in the economy, along with the stated policy adjustments, led to considerable changes in land use over the last 10 years. We anticipate that our results will be help policy makers to implement rational land use adjustment, providing insights on how to balance development with habitat conservation to help maintain wild Asian elephant populations.

Here, as a working hypothesis, we suggest that the relative policies and market trends that encouraged cash forest plantation have strong and negative effects on the distributions of Asian elephants^26^. If correct, several specific preconditions must hold: (1) the survival of Asian elephants depended on natural forests as their suitable habitats; (2) natural forests were affected negatively by the encroachment of cash forest (in this case primarily rubber and tea); (3) cash forest encroachment were stimulated by the demand for local economic development through relative policies and markets. Although we cannot directly test our working hypothesis through rigorous manipulative experiments, it is possible to derive reasonable inferences by collecting useful evidences that can reflect these preconditions across time series^26^.

If the above hypothesis relations hold, the pathway from polices and markets to Asian elephants should be supported by the following predictions: (1) the distributions and their trends of Asian elephants consisted with those of natural forests; (2) intense contradiction were showed between cash forest and natural forest, while the land use for cash forest was converted mainly from natural forests; (3) the preferences from policies and markets had similar trends with the development or recession of cash tree plantations (primarily rubber and tea). More specifically, in this paper, we studied regional changes in elephant distribution and human land use over the last 40 years, and collected evidences of policies and markets related to cash forest, with a strong focus on the last 10 years.

## Results

### Historical distributions and land cover types

**Figure 1:**
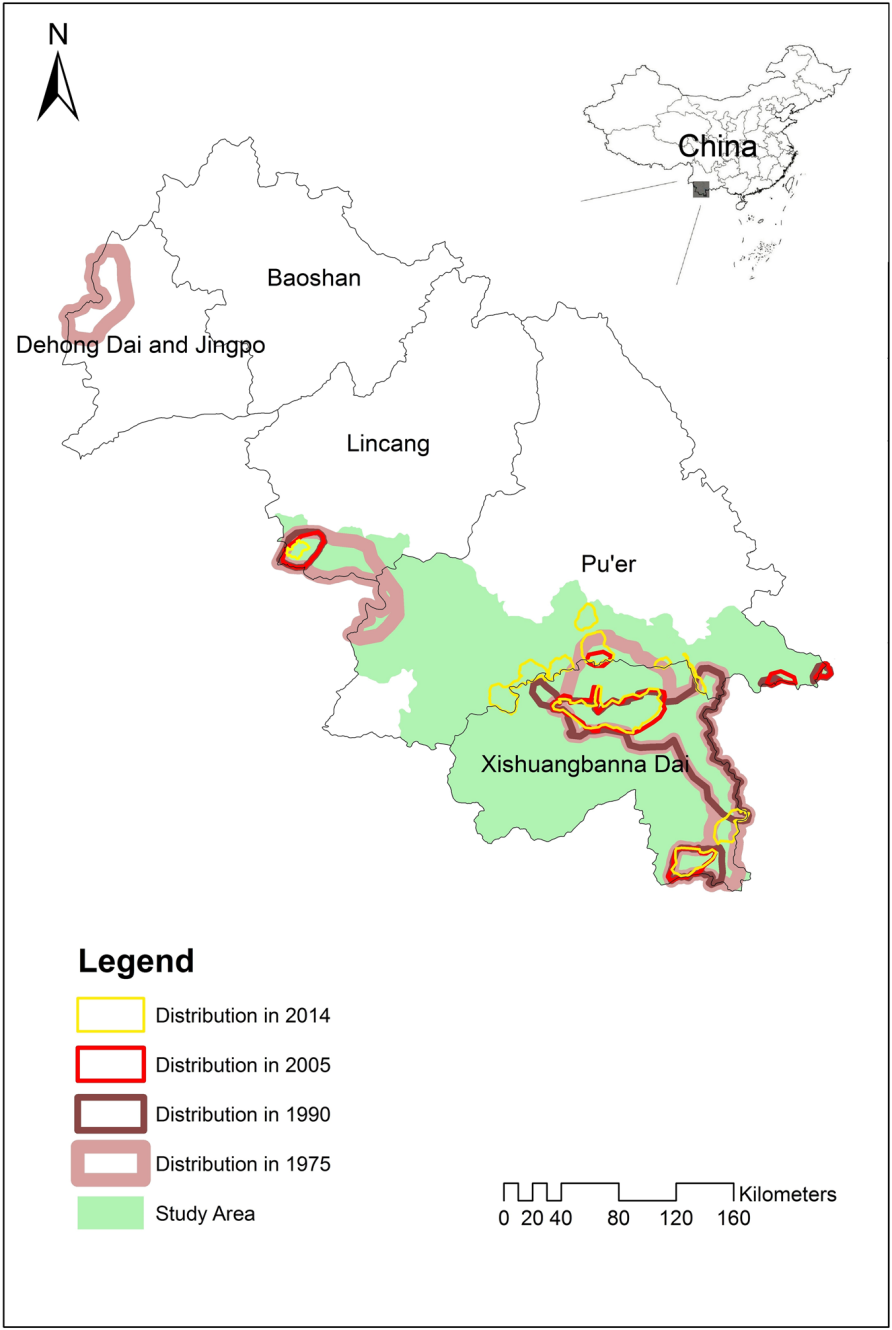
Distribtuion areas of Asian elephants in 1975, 1990, 2005 and 2014. ArcGIS 10.2 (ESRI Ltd., CA, USA. www.esri.com) were used to analyze the remote sensing images and GIS data.

We found that the distribution of Asian elephants has changed significantly over the last 40 years in China (Fig. 1). The population in Dehong disappeared entirely after 1975. Herds of elephants used to migrate more frequently near the border of Xishuangbanna and Pu’er, including the disappearance and appearance of a few small distribution areas. Overall, the distribution of elephants has shrunk, becoming highly fragmented.

**Figure 2:**
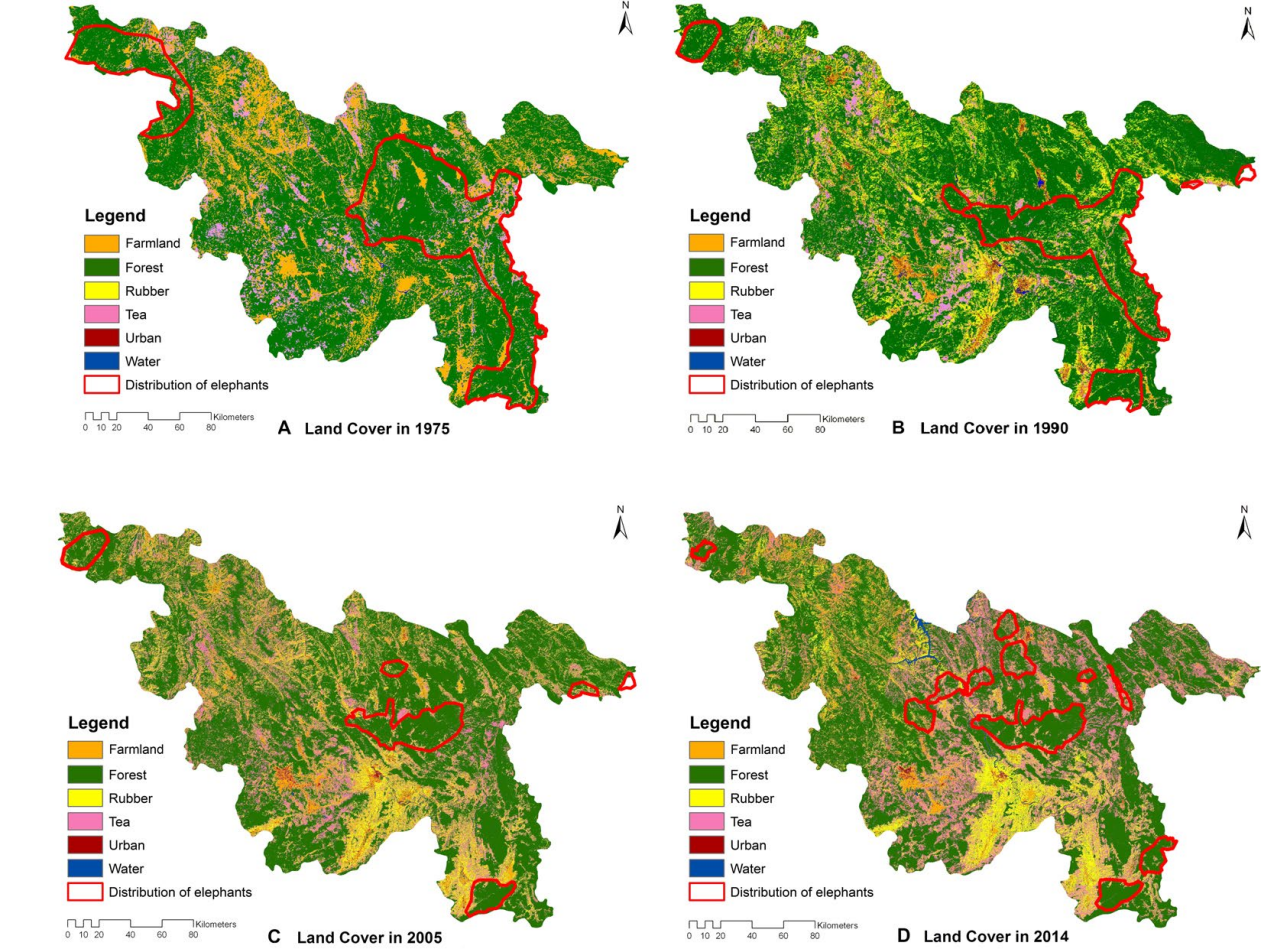
Land cover classification maps with related elephant distributions in 1975, 1990, 2005 and 2014. *Software: ArcGIS 10*.*2* (*ESRI Ltd*., *CA*, *USA*. www.esri.com).

**Figure 3:**
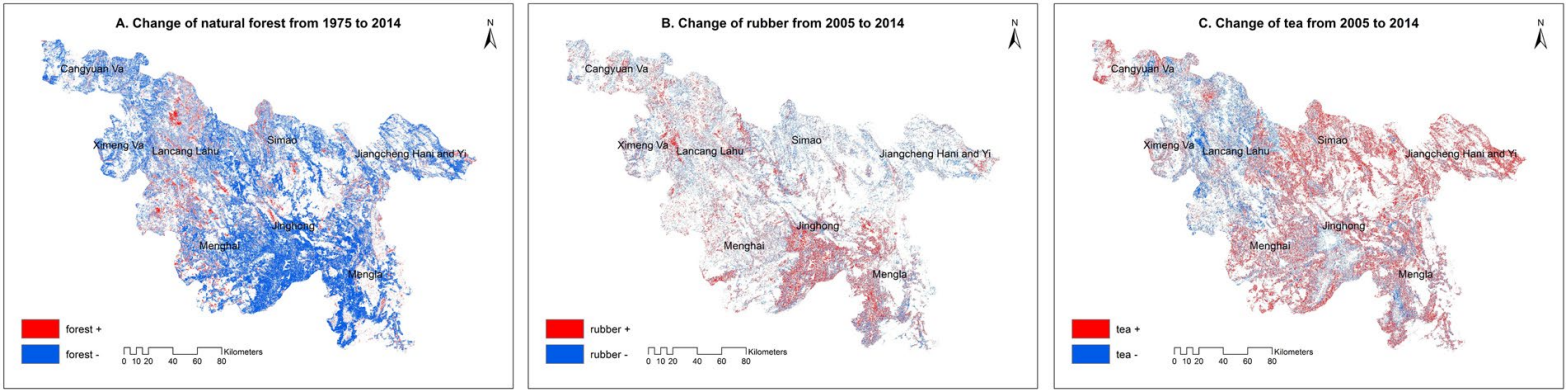
Spatial changes of different land cover (“+” means the appeared area; “−” means the disappeared area). (**A**) change of natural forest from 1975 to 2014; (**B**) change of rubber from 2005 to 2014; (**C**) change of tea from 2005 to 2014. *Software: ArcGIS 10*.*2* (*ESRI Ltd*., *CA*, *USA*. www.esri.com).

The land cover types compared with the distribution of Asian elephants showed that elephants primarily occupied continuous natural forests, and the distribution ranges of Asian elephants was similar as the distribution of unbroken natural forest, becoming shrunk and fragmented over the last 40 years (Fig. 2). However, natural forests were encroached obviously by rubber and tea. Over the last 40 years, the natural forest area decreased from 69.31% to 57.81%, while farmland area decreased from 21.13% to 6.45%. In contrast, rubber plantations expanded 23.4-fold, occupying 0.52% versus 12.71% of the study area in 1975 and 2014, respectively. The area covered by tea plantations also increased over the last 40 years, from 8.77% to 22.01%. The urban area increased 4.5-fold, from 0.11% to 0.51% (Table 1, the overall accuracy of classification was 78%). We mapped the change in forest cover from 1975 to 2014 (Fig. 3A). This map showed that the greatest forest loss has occurred in the southern part of our study area (including Menghai, Jinghong, and Mengla).

**Table 1:** Land cover types in Asia elephant range in China in 1975, 1990, 2005 and 2014 (km^2^).

Year	1975		1990		2005		2014		Total changes
	Area (km^2^)	Percentage	Area (km^2^)	Percentage	Area (km^2^)	Percentage	Area (km^2^)	Percentage	Area (km^2^)
Forest	26,785	69.31%	25,735	66.59%	24,087	62.08%	22,430	57.81%	−4355
Rubber plantation	202	0.52%	1,110	2.87%	4,961	12.79%	4,930	12.71%	4,728
Tea plantation	3,389	8.77%	7,829	20.26%	6,583	16.97%	8,539	22.01%	5,149
Farmland	8,165	21.13%	3554	9.20%	2,857	7.36%	2,504	6.45%	−5661
River and lake	63	0.16%	159	0.41%	97	0.25%	108	0.28%	44
Urban area	44	0.11%	260	0.67%	212	0.55%	200	0.51%	155
Total	38,647	100.00%	38,648	100.00%	38,648	100.00%	38,797	100%	150

### Land use conversion between 2005 and 2014

In addition to the loss of natural forests driven by the encroachment of cash forest, there were mutual competition between rubber and tea. The 2005 and 2015 land cover maps show that the area covered by tea plantations has markedly increased (Fig. 2C,D). Rubber plantations became concentrated in the south (Fig. 3B), while extensive forests and scattered rubber farms were converted to tea plantations, mainly in the middle and eastern areas (Fig. 3C).

We created a land use transfer matrix, which indicates the net transfer in the quantity of variable land use types between 2005 and 2014 (Table 2). This matrix shows current land use changes and the potential drivers of these changes. The area covered by rubber plantations, farmland, forests, and urban areas decreased, whereas the area covered by water bodies and tea plantation increased. Tea plantations showed the largest increase (1956 km^2^), which was mostly transferred from forests (1671 km^2^) and rubber plantations (229 km^2^). The area covered by forests noticeably decreased (−1,658 km^2^), and was primarily converted to tea plantation. Only a small area of rubber plantation (210 km^2^) was transferred to forest, with the area covered by rubber plantations remaining nearly stable.

**Table 2:** Land use transfer matrix from 2005 to 2014 in the Asian elephant range area in China (km^2^).

Year	Land cover types	Water	Tea plantation	Rubber plantation	Farmland	Forest	Urban area
2005	Total area	97	6,583	4,961	2,857	24,087	212
Land cover changes (km^2^)	Water	—	−30	−31	−27	−13	−6
	Tea plantation	30	—	−229	−77	−1,671	−10
	Rubber plantation	31	229	—	−403	210	−25
	Farmland	27	77	403	—	−165	10
	Forest	13	1,671	−210	165	—	19
	Urban area	6	10	25	−10	−19	—
	Total change	108	1,956	−41	−352	−1,658	−13
2014	Total area	205	8,539	4,920	2,504	22,430	200

### Policies and market trends for rubber and tea

**Figure 4:**
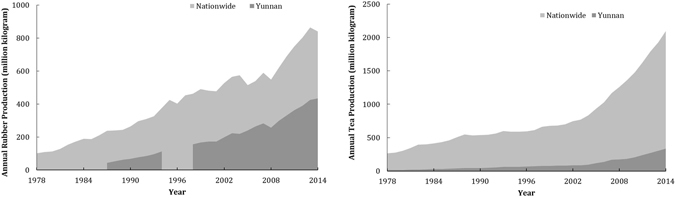
Annual yields for rubber and tea in Yunnan Province and nationwide during 1978 to 2014 (millon kilogram). (**A**) annual rubber yields (no data had been recorded for Yunnan from 1978–1987 and from 1995–1997) (**B**) annual tea yields.

**Figure 5:**
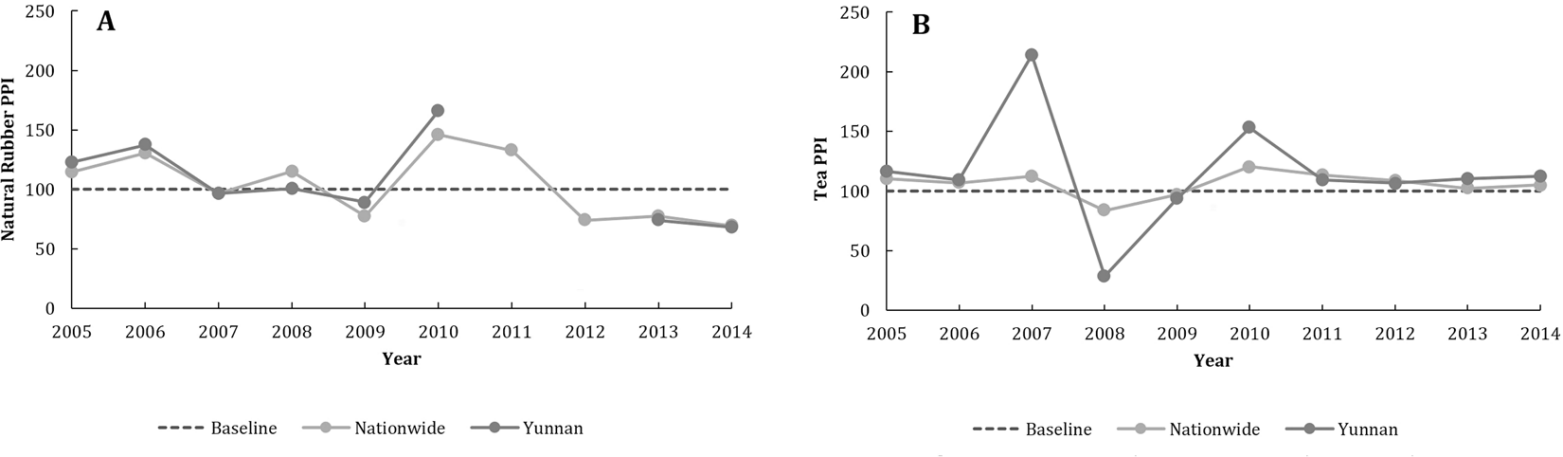
Annual Producer Price Index (PPI) for natural rubber and tea in Yunnan Province and nationwide during 2005 to 2014. (**A**) natural rubber PPI (**B**) tea PPI. Baseline: the price in the previous year is 100. (A Producer Price Index (PPI) measures the average changes in prices received by domestic producers for their output. In the US, the PPI was known as the Wholesale Price Index, or WPI, up to 1978).

Over the last 40 years, the annual yields of both the rubber and tea industries has shown a continuous increase (Fig. 4), with the trends in Yunnan Province reflecting those of the country as a whole. However, the Central Government initiated two major conservation policies in the late 1990s**:** the Natural Forest Conservation Program (NFCP) implemented in 1998, and the Sloping Land Conservation Program (SLCP) implemented in 1999^27^. Several local conservation activities were also initiated in 2000s primarily for the sustainable development of rubber^24, 25^. On the other hand, he annual PPI of rubber and tea fluctuated from 2005–2014 (Fig. 5). The changes in PPI reflected the continuous fall in the price of natural rubber (as low as 70), and the steady rise in tea prices (above 100) after 2011. Overall, the increase of tea and the halt of rubber during 2005–2014 could be inferred by the preferences of policies and markets.

## Discussion

### Distribution of elephants and land use changes over the last 40 years

Our study shows that the distribution ranges of Asian elephants have shrunk and have become increasingly fragmented, similar as the distribution of unbroken natural forest (Fig. 2). The current distribution area of Asian elephants represents less than one third of what it was in 1975. Both the continuous decline in forest cover and anthropogenic activity have restricted the movement of elephants, forcing them to forage in agricultural areas. As a result, elephants destroy crops, rubber, tea, and human lives, leading to human-elephant conflicts^28^.

The reduction in farmland cover detected by our study suggests a decline in traditional farming practices, with greater investment in tea and rubber cultivation. In our village interviews, elephants destroying crops was stated as the main reason for giving up traditional farming. In parallel, urban area have expanded 4.5-fold, because of economic development that is mostly attributed to the tea and rubber industries. The area covered by rubber plantations noticeably increased from 1990–2005, but stabilized during 2005–2014 (Table 1). The accuracy of historical land use classification was acceptable, and published literatures have also mapped the same continuous expansion of rubber plantations and decrease of natural forest in Xishuangbanna before 2005^10, 29^, but we found the halt of expansion during 2005–2014. In contrast, the area covered by tea plantations declined between 1990 and 2005, but further increased to 22.01% between 2005 and 2014.

These changes reflect policy guidance and market fluctuations. During 1990–2005, rubber cultivation was promoted by governmental support policies and large economic benefits, which led to land use conversion from forest and tea to rubber plantations^10, 30^. In recent years, there has been a move toward more sustainable development, including initiatives for the responsible development of rubber cultivation^31^. Rubber plantations and management were standardized and controlled, while illegal rubber plantations at high elevations and on steep slopes were converted back to forest^32^. These measures contributed to the stagnation and concentration of area used to cultivate rubber between 2005 and 2014 (Fig. 3B). In addition, because of the continuous fall in the price of natural rubber, and steady rise in tea prices after 2011 (Fig. 5), the greater profit accrued from tea, rather than rubber production, drove land use expansion in favor of tea cultivation.

### Impact of rubber and tea plantations on elephant habitats

Yunnan Province is the second largest base for natural rubber production in China, with Xishuangbanna being currently one of the most productive regions for rubber. Newly developed varieties of rubber plants may be planted at higher altitudes and on steeper slopes, compared to previous rubber plants^33^. Asian elephants might sometimes take refuge in rubber plantations, but they are not suitable habitats.

Rubber is an introduced species from the Amazon Basin. The expansion of rubber plantations significantly reduces the biodiversity of local species. Consequently, the ecosystem is weakened^33, 34^, which increases its vulnerability to disease, insect pests, and invasive species^35^. Rubber has a strong water absorption and retention capacity, prompting research efforts to address the potential effects of rubber plantations on water and soil loss and drought^36^. Furthermore, local rubber processing factories release waste gasses and water, which have damaged ecosystems in recent years^35^.

In contrast, tea cultivation is a traditional practice in Yunnan Province, and is believed to affect the ecosystem less than rubber plantations. However, the major expansion of the area used for tea area also threatens Asian elephants through habitat loss and fragmentation. In addition, the misuse of pesticides and herbicides on tea farms might lead to considerable pollution^37^. On occasion, tea plantations provide safe corridors for elephant groups moving from one habitat to another. However, various elephant-deterrent methods are employed that often hinder the passage of elephants, exemplifying the increasing human-elephant conflict^28^.

### Impact of conservation policies and market trends on land use

In China, changes to forest cover are considered to be driven by state policies, with the scope of directly targeted environmental policy reforms being arguably unprecedented^27^. Ancient swidden-fallow agriculture dominated many ethnic communities in China until the 1960s, when the Great Leap Forward was implemented to eliminate poverty. Extensive deforestation occurred to plant cash crops, such as rubber, before the nationwide Household Responsibility System was implemented in late 1970s. Furthermore, the collective forest tenure reform stimulated smallholder farms to follow the lead of state farms to clear cut old growth forests and to plan rubber trees. After the forestland allocation policy (“Forestry Three Fixed”) in 1982, land use decisions were further affected by the market. Large areas of fallow forests were converted into cash crops to raise the incomes of farmers. However, some natural hazards, such as the Yellow River Drought and the Yangtze River Flood, stimulated the state to initiate a range of forestry policies to conserve and reforest the nation, including the NFCP in 1998 and SLCP in 1999.

Yet, afforestation efforts often fail to promote biodiversity, due to their being focused on fast-growing or economically valuable species, such as rubber, walnut, and fruit trees. After 2003, high commodity prices, which were largely driven by increasing domestic demands, led to the massive expansion of rubber plantations. In addition, market demand and land-tenure reform encouraged capital investment and labor migration for the expansion of monoculture rubber plantations. However, since the price of rubber was at a record high in 2011, entrepreneurs from China chose to invest in rubber plantations in other countries, such as the less developed regions of Mekong, including Northern Thailand, Laos, and Myanmar^29^, leading to the downslide of domestic rubber.

Whether cash tree plantations should be included in reforestation has been a controversial issue. In the NFCP and SLCP programs, rubber forests were considered a substitute for natural forests. As a result, rubber was included in the state statistics as forest cover^30^, which allowed local governments to claim falsely that they were maintaining forest cover. Most cash trees provide ample economic benefits locally, but provide substantially limited forest ecosystem services for regional and global beneficiaries^38, 39^. Consequently, the benefits of the policies from the 1990s have been overestimated because of forestation practices using cash trees.

### Strategies for balancing habitat conservation and local development

Because of changes in land use due to the growing rubber and tea industries, elephant habitats are under greater pressure, and have become highly fragmented^20^. Therefore, we propose that effective policies for forest protection should be explored, and that ecological corridors should be established.

During 2005–2014, only 210 km^2^ of land was converted to forest because of local government reforestation policies. In contrast, more than 1,600 km^2^ of forest was converted to tea plantations in the same period. We suggest that more land that falls inside the distribution ranges of Asian elephants should be converted from rubber and tea plantations back to natural forests to restore historical elephant habitat. Such practices could be led by the government with support from conservation groups and the participation of local communities. Allowing for the booming market and the expansion of tea plantations over the last 10 years, there is an urgent need to plan the development of tea industry properly, especially in Xishuangbanna and the southern part of Pu’er, based on our mapping (Figs 2D and 3C), to guarantee sufficient habitats for elephant populations to persist.

Furthermore, because of the negative impacts of monoculture cash trees, the diverse land use systems practiced by smallholders might represent the most ecologically appropriate and culturally suitable means for promoting sustainable local economies and livelihoods^40^. Pilot projects for mixed agroforestry systems have been conducted inside and outside of nature reserves in Yunnan Province, along with scientific studies on eco-compensation for reforestation and mixed “jungle rubber”^25, 31^. Some regions with lower rubber product value, such as high elevations and steep slopes, could be converted back to forests^10, 31^. The establishment of an “ecological tea garden” presents a new way to reduce the negative ecological impacts of tea plantation with higher product value per unit area. In summary, it is possible to realize conservation goals without compromising income levels through an effective combination of regulation, household payments, and monitoring land use change.

**Figure 6:**
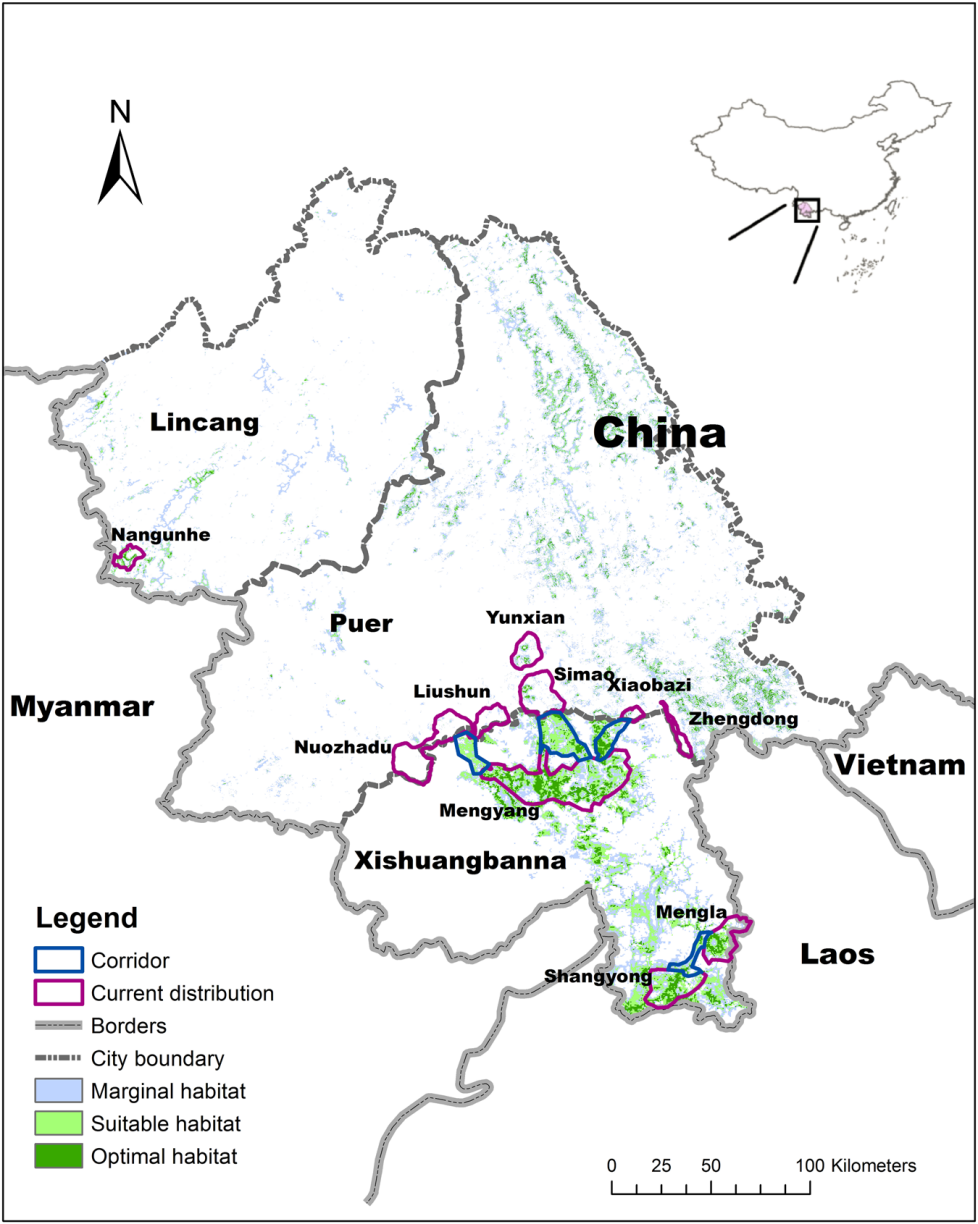
The expected design for Asian elephant corridors in China (Zhang *et al*.^8^). *Software: ArcGIS 10*.*2* (*ESRI Ltd*., *CA*, *USA*. www.esri.com).

Corridors could expand the range of suitable habitats by connecting isolated patches of land^41, 42^, reducing human-elephant conflicts, and increasing population size, including gene flow exchange^43, 44^. We propose establishing four corridors to link the highly fragmented natural habitat of elephants within the study area (Fig. 6), both by restoring existing natural forests and creating new habitats for elephants, based on our completed habitat valuation^8^. Because the proposed corridors will occupy land that has been autonomously used by local communities, the primary challenge in constructing the corridors is obtaining the cooperation of local people. Fortunately, a questionnaire indicated that 80.6% of local residents support the construction of corridors. The main concern of those opposing the corridors is the traditional concept of ethnic minorities and the economic benefits of local farmers. One compromise is a new planting pattern, in which plants that are both valuable economically and edible to elephants are established in and around corridors. The cash trees within the proposed corridors could be transitioned to a mixed agroforestry system, including secondary regeneration in the understory of rubber^10^. For example, tea could be intercropped with passionflower (*Passiflora caerulea*) or other fruit trees. Alternatively, plantations could support fast growing and high value tree species, such as *Osmanthus delavayi* and *Gmelina arborea*, as well as some medical species. Allowing for possible predation by elephants around corridors, a spatially-explicit, sustainable and risk-based insurance scheme was suggested through prioritizing the hotpots of human-elephant conflicts^45, 46^.

At present, the Chinese government is in the process of creating conservation corridors for Asian elephants that will connect four isolated national nature sub-reserves (Mengyang–Menglun–Mengla–Shangyong). These corridors will be created by replanting existing monoculture rubber plantations with native tree and shrub species. This plan is supported by the biodiversity conservation corridors initiative of the Asian Development Bank^47^. China and Laos have signed a Memorandum of Understanding to create cross-boundary protected areas to preserve wildlife habitat since 2009. A total of 200,000 hectares of cross-boundary biodiversity preservation region has been designated along the 220 km shared border between Xishuangbanna of China and Phongsali, Oudomxay, and Luang Namha of Lao PDR in 2014. Meanwhile, China is also seeking to establish trans-boundary elephant conservation opportunities with its counterpart in Myanmar. These initiatives bring hope to us for the long-term survival of elephants in the entire region.

In conclusion, we suggest that the relative policies and market trends related to cash forest have indirect effects on the distributions of Asian elephants. We anticipate that by mapping the spatial changes in the distribution of elephant habitat ranges versus that of rubber and tea plantations driven by policies and markets, local managers will be able to incorporate the needs of endangered elephants through creating space when planning plantations. Restoring elephant habitat and establishing ecological corridors are critical for the survival of elephants in this region; however, all stakeholders must benefit for such initiatives to be successful in the long-term.

## Methods

We used available documentation (local annals, statistic yearbooks, survey records of reserves, and published papers) and village interviews to map the distributions of elephants over the last 40 years. Vegetation samples and remote images of vegetation in the Asian elephant distribution areas were used to classify vegetation cover types and to analyze land use changes. Market data on natural rubber and tea were collected to interpret land use changes over the last 10 years when reforestation policies were practiced and the market changed.

### Study area

The study area encompassed Mengla County, Jinghong City, and Menghai County in Xishuangbanna; Jiangcheng County, Simao District, Lancang County, and Ximeng County in Pu’er; and Cangyuan County in Lincang. This area is located at the southwest edge of Yunnan Province between 21.14°–23.49° north latitude and 98.88°–102.36° east longitude, and covers a total area of 38,648 km^2^. The elevation in the study area ranges from 460 to 2600 m. The annual average temperature ranges from 17 °C to 21 °C, and annual average precipitation is approximately 1500 mm. Yunnan Province is one of the poorest regions in China because of its multi-ethnic culture and remote, mountainous geography. Agriculture and the collection of goods from the forest are the main economic sources here, with rubber and tea being widely cultivated in recent years for economic development.

### Asian elephant distributions and land use changes

Field data through direct survey to assess the distribution and population trends of elephants across its geographical range were limited. Data on the historical distribution of Asian elephants were collected from existing documentation about the historical occurrence of elephants in China (see Supplementary Table S1), combined with village interviews. Most existing documentation were based on indirect estimates (e.g. dungs, interview) because of the physical danger by this large-bodied wildlife, and local ecological knowledge (LEK) could represent an important source of information on species that are charismatic and easily identifiable or of socio-economic importance^48–50^. From July to November 2014, we interviewed individuals in villages located to areas close to the recorded elephant occurrence points and the potential habitats, and the village locations were recorded by GPS. The list of interviewed villages was obtained through existing documentation and respective reserve or wildlife management office. These interviews covered 155 villages within or bordering the historical distribution of elephants. Interview record forms (see Supplementary Table S2) were completed by 223 people, mostly aged around 50.

During the interview, we employed local participants to guarantee communicating effectively in Mandarin or other local ethnic minority languages. All questionnaires were recorded in Chinese. The interviewed villagers were initially selected based on some criteria: aged from 40 to 65 (because the earliest time was 1975), regular activities in local forests, awareness and experience of elephants. Actually, many local forest rangers and former hunter met these criteria. Interview staff remained neutral and avoided using leading questions, in order not to influence informant responses.

Historical distributions of Asian elephants for 1975, 1990, 2005, and 2014 were mapped in ArcGIS. First, we draw preliminary sketches based on the existing documentation (see Supplementary Table S1). Data of village interview were used to rectify the distributions.

For image analysis and classification of the vegetation, we downloaded original Landsat satellite image data for 1975, 1990, 2005, and 2014 from websites offering Landsat remote sensing images (http://landsat.usgs.gov/worldwide_reference_system_WRS.php). Six data frames were used corresponding to the Worldwide Reference System (WRS) coordinates; paths 139–141, rows 44 and 45 in WRS-1, and paths 129–131, rows 44 and 45 in WRS-2. First, the Landsat images were radiometrically calibrated^51, 52^, and the recorded voltage or digital quantitative value (DN) of the sensor was transformed into the absolute radiation brightness value (radiance). Next, atmospheric correction was performed, to eliminate atmospheric and reflection effects. Ground control points were selected to conduct geometric corrections, and images from each period were joined together to create a single representation of the research area. Meanwhile, field survey data on vegetation points were collected to supervise the classification of these remote sensing images. Sampling transects were designed under the guidance of local reserve managers, to ensure that all vegetation types were covered. A total of 2512 points were recorded, once every 500–1000 m. Images were supervisedly classified into land cover types using specified vegetation sample points: natural forest, rubber plantation, tea plantation, farmland (rubber and tea excluded), water body, and urban land. 75% of the recorded points were used to classify while 25% were used to validate the accuracy. Finally, a land use transfer matrix for 2005 to 2014 was generated.

### Natural rubber and tea market data

We collected data on yields and price to provide a better overview about rubber and tea markets. The annual rubber yields in Yunnan Province and nationwide during the period of 1978–2014 and the annual tea yields in Yunnan province and nationwide from 1978–2014 were collected from the National Bureau of Statistics of China (http://www.stats.gov.cn/english/). We determined the annual Producer Price Index (PPI) for natural rubber and tea during 2005–2014, based on documents from the National Bureau of Statistics of China and the settlement prices from Shanghai Futures Exchange (http://www.shfe.com.cn/en/). PPI measures the average changes in prices received by domestic producers for their output.

### Survey application hardware and data processing software

The following hardware was used in the field surveys: Canon S3 camera, Garmin GPS 60, and Dell Inspiron 640 m laptop computer. Microsoft Excel 2013 was used to store and analyze market trend data. ENVI 4.8 (Exelis Visual Information Solutions. Boulder, CO, USA. 2010. www.exelisvis.com), ERDAS IMAGINE 9.2 (Leica Geosystems, USA. 2008. www.hexagongeospatial.com), and ArcGIS 10.2 (ESRI Ltd., CA, USA. www.esri.com) were used to analyze the remote sensing images and GIS data.

## Electronic supplementary material

Supplementary information
