# Supplementary material for: Conflict between conservation and development: cash forest encroachment in Asian elephant distributions
Source: Sci Rep. 2017 Aug 3;7:6404. doi: 10.1038/s41598-017-06751-6 (PMC5543071; doi:10.1038/s41598-017-06751-6)
Supplement: Supplementary file 1 — Supplementary information [file 41598_2017_6751_MOESM1_ESM.pdf]

Conflict between conservation and development: cash forest encroachment in Asian elephant distributions

Peng Liu[1], Hui Wen[2], Franziska K. Harich[3], Changhuan He[1], Lanxin Wang[4], Xianming Guo[4*], Jianwei Zhao[4], Aidong Luo[4], Hongpei Yang[4], Xiao Sun[1], Yang Yu [1], Shaobo Zheng[1], Jing Guo[1], Li Li[5], Li Zhang[1*]

[1] Key Laboratory for Biodiversity Science and Ecological Engineering, Ministry of Education, College of Life Sciences, Beijing Normal University, Beijing 100875, China

[2] College of Urban and Environmental Sciences, Peking University, Beijing 100871, China

[3] Institute of Plant Production and Agroecology in the Tropics and Subtropics, University of Hohenheim, Stuttgart 70599, Germany

[4] Research Institute of Xishuangbanna National Nature Reserve, Jinghong, Yunnan 666100, China

[5] Wildlife Management and Ecosystem Health Center, Yunnan University of Finance and Economics, Kunming 650221, China

Supplementary Information

Table S1. Partial Chinese documents about historical distribution area of Asian elephant in China

Title	authors	Year
The elephants in southern Yunnan	Shou, Z. H., Gao, Y.T., Lu, C. K.	1959
Distribution and conservation of elephants in Yunnan	Mammal research group of 1st lab of the Yunnan institute of Zoology	1976
The present status, historical distribution and conservation of wild elephant in China	Gao, Y. T.	1981
Elephant herds in China	Huang, P. Y., Wang, J. H.	1983
Abundance, distribution and conservation of rare mammals in Xishuangbanna	Yang, D. H., Zhang, C. J.	1987
Habit and abundance of elephants in Yunnan	Yang, D. H., Zhang, J. R., Li, C.	1987
Present status of Asian elephant	Li, Y. J.	1998
Distribution changes of Asian elephants in Xishuangbanna during the past 40 years	Wu, J. L., Jiang, W. G., Hu, J. S., et al.	1999
Present status of Asian elephants in Xishuangbanna	Jiang, W. G., Li, Z. Q., Hu, T., et al.	1999
Survey report on the activities of Asian elephants in Jiangcheng (unpublished)	Lin, L.	2004
Survey report on the activities of Asian elephants in Lancang (unpublished)	Lin, L.	2004
Distribution and habitats of Asian elephants in China	Feng, L. M.	2005
Preliminary study on the population ecology of Asian elephants *(Elephas maximus)* in Shangyong reserve, Xishuangbanna, China	Zhu, W. Q.	2006
Population dynamics，structure and seasonal distribution pattern of Asian elephant (*Elephas maximus*) in Shangyong Protected Area, Yunnan, China	Zhang, L. T.	2008
Distribution, abundance, origin and habitat use of Asian elephant in Mengla reserve, Xishuangbanna	Chen, D. K.	2008

Selection and distribution of the habitats of Asian elephants and their impacts on the vegetation, in Mengyang reserve	Lin, L.	2009
Habitats suitability valuation and ecologic corridor design for Asian elephants in Xishuangbanna	Jin, Y. F.	2010
Asian elephants in China: estimating population size and evaluating habitat suitability	Zhang, L., Dong, L., Lin, L., et al.	2015

Table S2. Record form for village interview

Number	Contents	Record 1	Record 2	Record 3
1	Village name			
2	Date			
3	Time			
4	Name of interviewee			
5	Age of interviewee			
6	Direction elephants come			
7	Direction elephants leave			
8	Whether did elephants eat crops			
9	The number of elephants			
10	Occurrence frequency around village			
11	Disappearance time			
12	Duration of disappearence			
13	Reappearance time			
14	Whether did elephants ear crop during reappearance			
15	Reappearance frequency			
16	The number of elephants during reappearance			
17	Additional information			
